# Repeated intravitreal injections of antivascular endothelial growth factor in patients with neovascular age-related macular degeneration may increase the risk of ischemic optic neuropathy

**DOI:** 10.1186/s12886-019-1284-x

**Published:** 2019-12-30

**Authors:** Yu-Yen Chen, Pesus Chou, Yu-Fang Huang, Hung-Jen Chien, Yu-Chieh Wu, Chia-Chi Lee, Li-Ying Huang, Hsin-Hua Chen

**Affiliations:** 1 Department of Ophthalmology, Taichung Veterans General Hospital, 1650 Taiwan Boulevard Sect. 4, Taichung, 40705 Taiwan; 2 School of Medicine, National Yang-Ming University, Taipei, Taiwan; 3 Community Medicine Research Center and Institute of Public Health, National Yang-Ming University, Taipei, Taiwan; 4 School of Medicine, College of Medicine, Fu Jen Catholic University, New Taipei, Taiwan; 5 Division of Endocrinology and Metabolism, Department of Internal Medicine, Fu Jen Catholic University Hospital, New Taipei, Taiwan; 6 Department of Medical Education, Fu Jen Catholic University Hospital, New Taipei, Taiwan; 7 Department of Medical Research, Taichung Veterans General Hospital, Taichung, Taiwan; 8 Division of Allergy, Immunology, and Rheumatology, Department of Internal Medicine, Taichung Veterans General Hospital, Taichung, Taiwan; 9 Institute of Biomedical Science and Rong-Hsing Research Center for Translational Medicine, Chung-Hsing University, Taichung, Taiwan

**Keywords:** Intravitreal injection, Antivascular endothelial growth factor, Neovascular age-related macular degeneration, Ischemic optic neuropathy, Cohort study, Risk factor, National Health Insurance Research Database

## Abstract

**Background:**

Previous case reports have demonstrated the occurrence of ischemic optic neuropathy (ION) following intravitreal injections of antivascular endothelial growth factor (anti-VEGF). However, no previous studies have investigated the impact of injection numbers on the risk of ION. The aim of our study was to investigate whether repeated intravitreal injections of anti-VEGF would increase the risk of subsequent ION in patients with neovascular age-related macular degeneration (AMD).

**Methods:**

A population-based, retrospective cohort study using the Taiwan National Health Insurance Research Database was conducted from 2007 to 2013. Neovascular AMD patients receiving intravitreal injections of anti-VEGF during the study period were enrolled in the study cohort. Enrollees were divided into three groups according to the categorized levels of injection number (first level: < 10 times, second level: 10–15 times, and third level: > 15 times). Kaplan-Meier curves were generated to compare the cumulative hazard of subsequent ION among the three groups. Cox regression analyses were used to estimate crude and adjusted hazard ratios (HRs) for ION development with respect to the different levels of injection numbers. The confounders included for adjustment were age, sex, and comorbidities (diabetes, hypertension, hyperlipidemia, ischemic heart disease, and glaucoma).

**Results:**

In total, the study cohort included 77,210 patients. Of these, 26,520, 38,010, and 12,680 were in the first-, second-, and third-level groups, respectively. The Kaplan-Meier method revealed that the cumulative hazards of ION were significantly higher in those who had a higher injection number. After adjusting for confounders, the adjusted HRs for ION in the second- and third-level groups were 1.91 (95% confidence interval [CI], 1.32–2.76) and 2.20 (95% CI, 1.42–3.43), respectively, compared with those in the first-level group.

**Conclusions:**

Among patients with neovascular AMD, those who receive a higher number of anti-VEGF injections have a significantly higher risk of developing ION compared with individuals who receive a lower number of injections.

## Background

Neovascular age-related macular degeneration (AMD) is characterized by the proliferation of abnormal blood vessels (neovasculature) from the choroid and can cause macular edema and severe vision loss. Intravitreal injection of steroids has the effect of decreasing macular edema [1–6]. In addition, the intravitreal injection of antivascular endothelial growth factor (anti-VEGF) can inhibits vascular endothelial growth factor (VEGF) and leads to marked decreases in neovascularization of both the choroid and retina [7–10]. Thus, intravitreal injection of anti-VEGF has been widely performed to treat neovascular AMD.

Ischemic optic neuropathy (ION), resulting from an insufficient blood supply, is the most common type of acute optic neuropathy in older patients [11, 12]. ION is classified as anterior or posterior ION according to the affected segment of the optic nerve. Both types are further categorized into arteritic (related to vasculitis), and nonarteritic (not related to vasculitis). Of these, nonarteritic anterior ION (NAION) is the most common type. The risk factors for NAION include a crowded optic disk, diabetes, hypertension, hyperlipidemia, and ischemic heart disease [13–17]. Diminished ocular blood perfusion due to elevated intraocular pressure (IOP) or glaucoma can also lead to the occurrence of ION [18–23].

Although intravitreal injection of anti-VEGF is effective for treating neovascular AMD, it may have some possible complications such as endophthalmitis, intraocular inflammation, IOP elevation, and ocular hemorrhage [24]. In addition, one rare ocular adverse effect is ION [25–27]. The underlying pathogenesis of the association between anti-VEGF and the risk of subsequent ION is still not fully known. One potential mechanism may be transient IOP elevation after intravitreal injection of anti-VEGF. Another possible explanation may be due to the properties of anti-VEGF. Anti-VEGF not only inhibits the formation of the neovasulature from the choroid, but also might result in inadvertent regression of the collateral vessels of the optic nerve head. Thus, increasing the number of injections may increase the risk of developing ION. To the best of our knowledge, no previous studies have investigated the impact of injection numbers on the risk of ION. The lack of evidence-based studies results from the rare occurrence of ION. In addition, systemic diseases such as diabetes, hypertension, hyperlipidemia, ischemic heart disease, and glaucoma, may confound the association between anti-VEGF and ION, and thus must be adjusted.

We conducted this study based on the Taiwan National Health Insurance Research Database (NHIRD) to elucidate the association between the intravitreal injection of anti-VEGF and subsequent ION. Our hypothesis is that a higher number of intravitreal injections of anti-VEGF will increase the risk of subsequent ION among neovascular AMD patients. We used the whole population database and therefore had large numbers of patients as well as a high level of statistical power. The completeness of the database ensures that the diagnoses of each patient are available, and the diagnoses were made according to the generally accepted International Classification of Diagnoses, Ninth Revision, Clinical Modification (ICD-9-CM) Codes.

## Methods

### Data source

The NHIRD was derived from the Taiwan National Health Insurance Program, which covers more than 99% of Taiwan’s 23 million residents. The NHIRD contains all the original claims data from the program and is released annually by the National Health Research Institute in an electronically encrypted form. The NHIRD consists of diagnoses, medical prescriptions, surgical procedures, and insurance registries and was released for scientific research after personal identification numbers were encrypted. This study was approved by the institutional review board of National Yang-Ming University Hospital (2015A018).

### Study subjects

We conducted a retrospective cohort study from January 1, 2007, to December 31, 2013. First, we selected patients from the NHIRD who were diagnosed with neovascular AMD (ICD-9-CM code 362.52) during the study period, which required confirmation by fundoscopy, fluorescein angiography (FA), and/or optical coherence tomography (OCT). In cases of neovascular AMD cases, FA can detect the presence of lesion and determine the size and location of the choroidal neovasculature. OCT can define the cross-sectional architecture of the retina and can reveal the presence of subretinal and intraretinal fluid. Patients diagnosed with neovascular AMD before the end of 2006 were excluded to ensure that all enrolled patients had new-onset neovascular AMD. If patients were found to have an obvious choroidal neovasculature and fluid in fundoscopy/FA/OCT, they were treated with intravitreal anti-VEGF. Those who received intravitreal anti-VEGF injections for the treatment of neovascular AMD were included in the study cohort. During the follow-up period, fundoscopy, FA, and OCT were performed regularly. If the neovasculature and fluid resolved and if no relapse occurred, the treatment with injections was ended. According to the number of anti-VEGF injections, patients in the study cohort were further divided into three groups (first level: < 10 times, second level: 10–15 times, and third level: > 15 times).

### Outcome variable

Patients in the study cohort were followed to identify the onset of ION (ICD-9-CM codes 377.41). Those with ION before the injection of anti-VEGF were excluded to ensure that all cases were new-onset ION.

### Demographic variables and comorbidities

Demographic variables such as age and sex were extracted from the database. Age was categorized into three levels: the first (< 60 years), the second (60–75 years), and the third (≥ 75 years) levels. Risk factors for ION such as diabetes (ICD-9-CM Codes 250), hypertension (ICD-9-CM Codes 401–405), hyperlipidemia (ICD-9-CM Codes 272), ischemic heart disease (ICD-9-CM Codes 410–414), and glaucoma (ICD-9-CM Codes 365) may confound the relationship between anti-VEGF and ION. Therefore, these comorbidities were identified in the medical records and included as covariates in our analyses.

### Statistical analysis

The Characteristics of the study cohort are presented according to age, sex, and comorbidities (e.g., diabetes, hypertension, hyperlipidemia, ischemic heart disease, and glaucoma). Group differences (first level: < 10 times, second level: 10–15 times, and third level: > 15 times) in these variables were analyzed by ANOVA tests (for continuous variables) and chi-square tests (for categorical variables). Then, the study cohort was followed until the occurrence of ION, dropout from the database, or the end of 2013, whichever came first. Survival analysis using the Kaplan-Meier method with the log-rank test was applied to describe and compare the cumulative hazard curves of ION according to the different levels of injection number.

Subsequently, all these variables (age, sex, number of intravitreal anti-VEGF injections, and comorbidities) were included in the Cox regression analyses. Unadjusted hazard ratios (HRs) for ION were computed according to each variable in the univariate analyses. Then, adjusted HRs for ION were derived from the multivariate analyses. Comorbidities were regarded as time-dependent variables. All statistical operations were performed using the SAS statistical package, version 9.2 (SAS Institute, Cary, NC, USA).

## Results

### Demographic and clinical characteristics of the study sample

In total, 77,210 patients were enrolled in the study cohort. Of them, 26,520 were in the first-level group, 38,010 were in the second-level group, and 12,680 were in the third-level group. Table 1 presents the demographic and clinical characteristics of the patients. The mean age in the study cohort was 67.4 years. The male to female ratio was 1.7:1. The prevalence of comorbidities was 35.6% for diabetes, 64.6% for hypertension, 44.3% for hyperlipidemia, and 29.8% for ischemic heart disease. Additionally, 16.5% of patients in the study cohort had glaucoma. The mean number of injections among the study cohort was 12.0, with a standard deviation (SD) of 2.9. Almost 50% of the patients had injection numbers in the range of 10 to 15. Significant differences were found across the three groups (first level: < 10 times, second level: 10–15 times, and third level: > 15 times) in age, sex, and comorbidities. The follow-up period in the study cohort was 3.50 ± 1.86 (mean ± SD) years, and did not differ significantly among the three groups. Of the 77,210 patients, 180 (0.23%) had a subsequent occurrence of ION: 40 (0.15%) in the first-level group, 100 (0.26%) in the second-level group, and 40 (0.32%) in the third-level group.

**Table 1:** Characteristics of the study cohort

Variable	Study cohort (*n* = 77,210)	First level (*n* = 26,520)	Second level (*n* = 38,010)	Third level (*n* = 12,680)	*p*-value
Age, years	67.4 ± 12.2	65.3 ± 13.0	68.1 ± 12.0	69.5 ± 10.5	< 0.0001
Age group, categorical					< 0.0001
< 60	20,020 (25.9)	8840 (33.3)	8940 (23.5)	2240 (17.7)	
60–75	32,780 (42.5)	10,400 (39.2)	16,410 (43.2)	5970 (47.1)	
≥ 75	24,410 (31.6)	7280 (27.5)	12,660 (33.3)	4470 (35.2)	
Sex					< 0.0001
Male	48,570 (62.9)	15,480 (58.4)	24,490 (64.4)	8600 (67.8)	
Female	28,640 (37.1)	11,040 (41.6)	13,520 (35.6)	4080 (32.2)	
Number of injections, times	12.0 ± 2.9	8.7 ± 3.4	12.3 ± 1.7	18.1 ± 4.2	< 0.0001
Number of injections, categorical					
First level (< 10)	26,520 (34.4)	26,520	0	0	
Second level (10–15)	38,010 (49.2)	0	38,010	0	
Third level (≥15)	12,680 (16.4)	0	0	12,680	
Diabetes					< 0.0001
Yes	27,490 (35.6)	10,770 (40.6)	12,510 (32.9)	4210 (33.2)	
No	49,720 (64.4)	15,750 (59.4)	25,500 (67.1)	8470 (66.8)	
Hypertension					< 0.0001
Yes	49,900 (64.6)	16,940 (63.9)	24,420 (64.2)	8540 (67.3)	
No	27,310 (35.4)	9580 (36.1)	13,590 (35.8)	4140 (32.7)	
Hyperlipidemia					< 0.0001
Yes	34,200 (44.3)	11,760 (44.3)	16,480 (43.4)	5960 (47.0)	
No	43,010 (55.7)	14,760 (55.7)	21,530 (56.6)	6729 (53.0)	
Ischemic heart disease					< 0.0001
Yes	22,990 (29.8)	7630 (28.8)	11,350 (29.9)	4010 (31.6)	
No	54,220 (70.2)	18,890 (71.2)	26,660 (70.1)	8670 (68.4)	
Glaucoma					< 0.0001
Yes	12,730 (16.5)	4570 (17.2)	5890 (15.5)	2270 (17.9)	
No	64,480 (83.5)	21,950 (82.8)	32,120 (84.5)	10,410 (82.1)	
Follow-up period, years	3.50 ±1.86	3.51 ±1.87	3.49 ±1.86	3.53 ±1.84	0.09
Incident ION	180 (0.23)	40 (0.15)	100 (0.26)	40 (0.32)	< 0.01

*ION* ischemic optic neuropathy. Data are presented as mean ± standard deviation or n (%)

### Kaplan-Meier curves and log-rank test

**Fig. 1:**
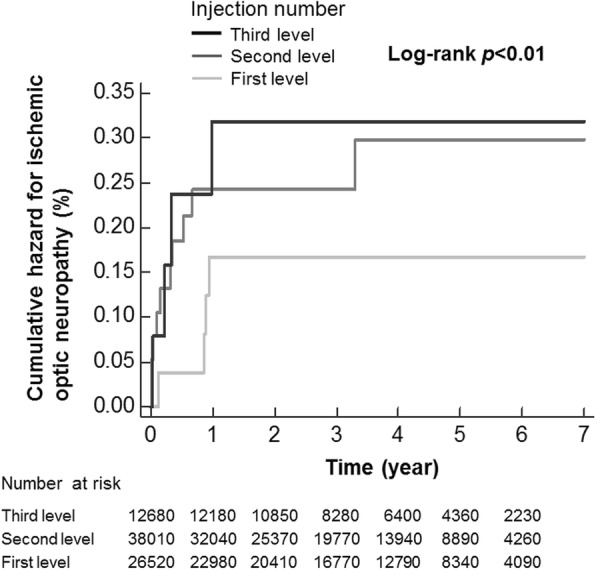
Kapan-Meier curves for ischemic optic neuropathy among neovascular AMD patients who received intravitreal injections of anti-VEGF. Level 1 represents less than 10 times; level 2 represents 10 to 15 times, and level 3 represents more than 15 times

Figure 1 illustrates the Kaplan-Meier curves of ION corresponding to each level of injection number. According to the log-rank test, the difference in cumulative hazards was significant (*p* < 0.01).

### ION risk

Table 2 displays the HRs for ION with regard to age, sex, injection number, and comorbidities. In the univariate Cox regression analysis, the second-level (10–15 times) and the third-level (≥ 15 times) groups yielded unadjusted HRs for ION of 1.75 (95% confidence interval [CI], 1.21–2.53) and 1.99 (95% CI, 1.28–3.08), respectively, compared with the first-level (< 10 times) group. The adjusted HRs obtained by comparing the second- and third level groups with the first-level group of were 1.91 (95% CI, 1.32–2.76) and 2.20 (95% CI, 1.42–3.43), respectively. Diabetes significantly increased the risk for ION in both the univariate and multivariate analyses. However, age, sex, hypertension, hyperlipidemia, and ischemic heart disease were not significant risk factors for ION in either the univariate or multivariate analyzes. Glaucoma increased the risk for ION in the univariate analysis, but the statistical significance was only marginal (unadjusted HR = 1.43; 95% CI, 1.01–2.03), and there was no significant difference in the multivariate analysis (adjusted HR = 1.35; 95% CI, 0.95–1.92).

**Table 2:** Risk Factors for ION in the study cohort

Variables	Univariate analysis		Multivariate analysis	
	Unadjusted HR (95% CI)	*p*-value	Adjusted HR (95% CI)	*p*-value
Age group, years				
< 60	Reference		Reference	
60–75	1.02 (0.76–1.39)	0.86	1.01 (0.75–1.38)	0.91
≥ 75	1.17 (0.86–1.61)	0.31	1.09 (0.76–1.58)	0.62
Sex (male vs. female)	0.84 (0.65–1.09)	0.19	0.82 (0.63–1.07)	0.14
Number of injections				
First level (< 10)	Reference		Reference	
Second level (10–15)	1.75 (1.21–2.53)	< 0.01	1.91 (1.32–2.76)	< 0.001
Third level (≥15)	1.99 (1.28–3.08)	< 0.01	2.20 (1.42–3.43)	< 0.001
Diabetes	1.82 (1.36–2.44)	< 0.0001	2.03 (1.46–2.81)	< 0.0001
Hyspertension	1.05 (0.83–1.35)	0.65	1.04 (0.85–1.30)	0.65
Hyperlipidemia	1.27 (0.95–1.70)	0.11	1.04 (0.75–1.43)	0.83
Ischemic heart disease	1.16 (0.85–1.58)	0.35	1.20 (0.86–1.67)	0.29
Glaucoma	1.43 (1.01–2.03)	< 0.05	1.35 (0.95–1.92)	0.09

*ION* ischemic optic neuropathy, *HR* hazard ratio, *CI* confidence interval

In the multivariable analysis, all other variables in the table were included for adjustment

## Discussion

This study is the first to reveal the increased risk of ION after repeated injections of anti-VEGF among neovascular AMD patients. In our population-based study, utilizing Taiwan’s NHIRD with a long (7-year) study period, we found that among patients with neovascular AMD, the risk of ION significantly increased in those who received more anti-VEGF injections, after adjusting for confounders.

AMD is a multifactorial disease, and advanced age is a main predisposing factor. In our study, the study cohort had a mean age of 67.4 years, and nearly one-third of the patients were older than 75 years. These results were compatible with a previous hospital-based study regarding anti-VEGF use among neovascular AMD patients in Taiwan [28]. It is noteworthy that the distribution of comorbidities was higher in our study cohort than in those without AMD in another population-based study in Taiwan [29]. In addition, Table 1 revealed significant differences in age, sex, and comorbidities according to the number of injections. Therefore, the group differences in these variables were adjusted in our subsequent Cox regression analyses.

The Kaplan-Meier curves with the log-rank test (Fig. 1) and Cox regression analyses (Table 2) in our study revealed that a higher number of injections was associated with a higher risk of subsequent ION. Very few case reports have described the onset of ION following the intravitreal injection of anti-VEGF. Hosseini et al. reported a 72-year-old woman with NAION occurring 1 week after the second intravitreal injection of anti-VEGF under the indication of active subfoveal choroidal neovascularization [25]. In their 2009 case report, Ganssauge et al. presented a 51-year-old man with pseudoxanthoma elasticum who received an intravitreal injection for choroidal neovascularization secondary to angioid streaks. Two weeks later, NAION was observed [26]. Huang et al. described a case of a 38-year-old woman with diabetic retinopathy. Three weeks after intravitreal injection of anti-VEGF, anterior ION occurred [27]. Although the elevated IOP that occurs during intravitreal injection might have a compression effect on the optic nerve head, the three patients with ION did not have an elevated IOP. It is possible that the IOP elevation was temporary and was not detected, or it is possible that other pathogeneses, such as anti-VEGF itself, precipitated ION.

Previous studies have shown that VEGF plays a role in modulating both vascular tone and blood flow autoregulation [30]. Ameri et al. found that a sudden decrease in the effective VEGF concentration might be responsible for the closure of normal capillaries [31]. Therefore, anti-VEGF may diminish the blood perfusion to the optic nerve head and cause ION. Additionally, VEGF has been reported to have both neurotrophic and neuroprotective effects [32]. In a diabetic rat model following intravitreal anti-VEGF injection, the rate of apoptosis increased in retinal ganglion cells [33]. It is possible that the optic nerve head is also directly disturbed by anti-VEGF and that the influence is additive after repeated injections. Therefore, repeated injections of anti-VEGF may lead to a higher risk of ION.

One limitation of our study is the lack of visual acuity and IOP values in our NHIRD. In addition, we retrieved patients receiving anti-VEGF injections through the procedure codes, but we could not differentiate what kind of anti-VEGF the patients received. These are the inherent drawbacks of our database. Further clinical studies are necessary in order to include these information.

Another limitation of our study is that we lacked controls with similar demographics. More studies regarding the comparison of ION risk between those with anti-VEGF and matched controls (without anti-VEGF) are warranted. In our study, patients who underwent fewer than 10 injections (the first-level group) were regarded as the reference group. However, the first-level, second-level, and third-level groups did not have similar baseline characteristics. Patients in the groups with higher numbers of intravitreal anti-VEGF injections were significantly older and had a significantly higher prevalence in hypertension, hyperlipidemia, and ischemic heart disease. These systemic factors may have affected the incidence of ION. Additionally, older people tend to have more comorbidities including diabetes, hypertension, and ischemic heart disease, which may coexist with ION. These confounding effects potentially complicated our analyses. We tried as possible as we could to adjust these factors in the Cox regression. However, from the perspective of epidemiology, not all confounders were measurable and could be adjusted with statistical methods. Therefore, randomized control trials are still needed to address this issue.

Our study has the strengths of a large sample size, an extended study period, a statistical analysis that adjusted for confounders, and validated diagnoses. In our health care system, the National Health Administration (NHA) frequently checks the cross-consistency of claims and chart data. The NHA also confirmed diagnoses that have been approved by a standard protocol of examinations. Therefore, the diagnoses in our database have a high degree of accuracy. It is noteworthy that in the diagnosis of neovascular AMD, OCT plays an important role. Although the brand or type of OCT machine is not exactly the same in every hospital or clinic, these machines are all useful for detecting the neovasculature and fluid. Some newer modalities, such as spectrum domain OCT, swept source OCT, enhanced depth OCT, and OCT angiography can provide earlier detection of the choroidal neovasculature and can be applied in additional research.

We cannot conclude a causal relationship between repeated anti-VEGF injections and ION. Most likely, the observation that a higher incidence of ION in patients receiving more anti-VEGF injections only reflects a greater need for anti-VEGF in those patients who tend to have ION. At present, all analyses were based on our database, and we derived a positive association between repeated anti-VEGF injections and ION. The underlying mechanism of the association is still not fully understood. Further basic research, animal models, and possibly large-scale prospective cohort studies are needed to unravel the pathogenesis.

## Conclusions

Currently, intravitreal injection of anti-VEGF has become the mainstay of treatment in neovascular AMD. Our study, revealed the possible complication of ION after repeated injections. Our recommendation is not to oppose treatment or change the protocols, which require more time and additional studies. At present, our study is simply a reminder for ophthalmologists to check optic nerve changes following anti-VEGF injections among neovascular AMD patients, especially those who receive a high number of injections. The risk of ION should also be considered when determining the use of anti-VEGF injections for the treatment of neovascular AMD.

## Data Availability

The datasets used and/or analysed during the current study are available from the corresponding author on reasonable request.

## Abbreviations

AMD: Age-related macular degeneration
anti-VEGF: Antivascular endothelial growth factor
CI: Confidence interval
FA: Fluorescein angiography
HRs: Hazard ratios
ICD-9-CM Codes: International Classification of Diagnoses, Ninth Revision, Clinical Modification Codes
ION: Ischemic optic neuropathy
IOP: Intraocular pressure
NAION: Nonarteritic anterior ION
NHA: National Health Administration
NHIRD: National Health Insurance Research Database
OCT: Optical coherence tomography
SD: Standard deviation
VEGF: Vascular endothelial growth factor
